# Pathogenesis and Causes of Premature Ovarian Failure: An Update 

**Published:** 2011-09-23

**Authors:** Mahbod Ebrahimi, Firoozeh Akbari Asbagh

**Affiliations:** 1 Obstetrics and Gynecology Department, Women’s (Mirza Koochak Khan) Hospital, Faculty of Medicine, Tehran University of Medical Sciences, Tehran, Iran; 2 Obstetrics and Gynecology Department, Faculty of Medicine, Bushehr University of Medical Sciences, Bushehr, Iran

**Keywords:** Premature Menopause, Premature Ovarian Failure, Hypergonadotropic Ovarian Failure, Hypergonadotropic Hypogonadism, Premature Ovarian Insufficiency

## Abstract

Premature ovarian failure (POF) affects 1% of young women. This condition has significant psychological sequelae and major health implications. POF seriously interferes with fertility and family planning. Diverse etiologies are associated with POF. Literature review related to the causes and pathogenesis of POF, cited between the year 1900 and May 2010. POF may be either spontaneous or induced. The known causes include:

- Genetic disorders, which could involve the X chromosome or autosomes. However, the growing body of literature demonstrates a list of newly discovered mutations that may be responsible for causing POF. Most of these mutations are extremely rare, and most cases of POF are still considered to be idiopathic.
- Autoimmune causes; there is some evidence of an association of POF with lymphocytic oophoritis and other autoimmune disorders. Antiovarian antibodies are reported in POF, but their specificity and pathogenic role are obscure.
- Iatrogenic causes; chemotherapy, radiotherapy and pelvic surgery can lead to POF.
- Infectious Causes; some viral and microbial infections can be followed by POF.
- Environmental toxins, such as cigarette smoking are reported as risk factors of spontaneous POF.
- Idiopathic; in most cases, no identifiable etiology can be recognized after complete evaluation.

## Introduction

### Definition and incidence

Premature ovarian failure (POF) is a mysterious disorder. It is defined by the association of amenorrhea, sex steroid deficiency and elevated (menopausal) levels of serum gonadotropins before the age of 40 years. It is not a rare condition; its incidence is estimated to be as great as 1 in 100 by the age of 40, and 1 in 1000 by the age of 20 years (1-5).

In women with primary amenorrhea, the prevalence is 10-28% and in those with secondary amenorrhea, POF occurs in 4-18% of patients (2, 4, 5). This condition has significant psychological sequelae and major health implications (6). POF seriously interferes with fertility and family planning (7, 8).

At one time, POF had been considered irreversible on the basis of early studies, which suggested that a serum follicle stimulating hormone (FSH) level of more than 40 IU/ L is associated with permanent cessation of ovarian function due to ovarian follicle depletion (9). The notion of permanent cessation of ovarian function was challenged in later reports (10). Intermittent ovarian function and even spontaneous pregnancy have been reported in young women with spontaneous POF subsequent to diagnosis (4, 7, 8). So ''premature ovarian failure'' is a misnomer. ''Premature ovarian dysfunction'' or ''premature ovarian insufficiency'' may be a more accurate phrase to reflect the reversible nature of this condition, and avoid the negative connotation that comes with the term ''failure'' (11, 12).

Although most cases of POF are idiopathic, with no identifiable etiology even after a thorough evaluation (13), diverse etiologies have been associated with POF: genetic aberrations, autoimmune ovarian damage, iatrogenic factors, infectious agents, toxins and environmental factors (Table 1).

**Table 1:** Etiological factors of premature ovarian failure


**1. Idiopathic causes**
**2. Genetic disorders**
X chromosomal abnormalities
X monosomy (Turner’s syndrome)
X Trisomy
X- autosomal translocation
X- chromosomal deletion
Fragile X syndrome
Mosaic karyotype
Bone morphogenetic protein 15 (BMP15) gene mutation
Autosomal disorders
Galactosemia
Blepharophimosis- ptosis- epichanthus inversus syndrome (BPES)
Autoimmune polyendocrinopathy- candidiasis-ectodermal dystrophy (APECED)
Steroidogenic enzyme defect
Gonadotropin receptor dysfunction
Inhibin gene mutation
Noggin mutation
**3. Autoimmunologic causes **
**4. Iatrogenic causes**
Pelvic surgery
Irradiation
Chemotherapy
**5. Infectious Causes**
Varicella
Mumps
Cytomegalovirus
Tuberculosis
Malaria
Shigella
**6. Environmental toxins**


To the best of our knowledge, this study is the first review article about POF in Iranian literature. This review presents the causes and pathogenesis related to POF, obtained by Medline, Pub Med, Google Scholar, the Cochrane Library, Iran Medex and hand searches of pertinent references of English literature on POF, cited between the year 1900 and May 2010.

### Causes of POF

#### Genetic disorders

The observation of familial cases with POF indicates the role of genetic aberrations in its pathogenesis (14). Although genetic defects mostly involve the X chromosome, an increasing number of studies have also documented the involvement of autosomal chromosomes. A growing body of literature demonstrates a list of newly discovered mutations that can cause ovarian failure. Most of these mutations are extremely rare, and most cases of POF are still considered to be idiopathic.

Various genetic mechanisms implicated in the pathogenesis of POF include reduced gene dosage and non-specific chromosome effects that impair meiosis. These can lead to ovarian failure by causing a decrease in the pool of primordial follicles, increased atresia of the ovarian follicles due to apoptosis, or failure of follicle maturation (15).

#### X chromosomal abnormalities

Genetically, ovarian failure is associated with X chromosomal abnormalities. These abnormalities could include a small defect in chromosomal arrangement such as deletions, isochromosomes and balanced X chromosome-autosomal translocations. However, complete deletion of one X (Turner’s syndrome) has also been recorded (16).

#### X monosomy (Turner’s syndrome)

Complete or near-complete absence of one X chromosome is the most common chromosomal defect in humans (17). This condition leads to ovarian dysgenesis characterized by primary amenorrhea, short stature and characteristic phenotypic features. Two functioning X chromosomes are necessary for normal ovarian function (18). In the presence of only one X chromosome, ovarian follicles degenerate from birth onwards. This is most likely caused by lack of diploid dosage of one or more vital genes and accelerated follicular atresia. Histological data indicate that oogenesis proceeds normally in these individuals until diplotene oocytes begin to be incorporated into follicles. There is a subsequent block in the production of complete follicles manifesting as follicular atresia. In 80% of cases, the paternally derived is lost (19).

However, the identification of genes or critical regions responsible for individual Turner’s syndrome features has turned out to be problematic. Cytogenetic data indicate the reduced dosages of genes on the short arms of the X (Xp) and Y (Yp) chromosomes (2. 6Mb Xp-Yp pseudoautosomal region) tends to be associated with short stature and somatic anomalies (20, 21), whereas deletions on the long arm of the X (Xq) chromosome tends to be associated with ovarian failure but no somatic anomalies (22, 23). The degrees of ovarian dysfunction and the extent of the somatic anomalies are variable. While the most affected patients have primary amenorrhea, streak gonads and absence of pubertal development, some develop normally and occasionally have a successful pregnancy and then secondary amenorrhea, while other patients have no somatic defects.

Association between trisomy X and POF have been reported (24). In one reported series, 3. 8% of patients with POF had the triple X syndrome (25). POF has also been reported in a girl with 48XXXX (26). Individuals with 45X/46XX and 46XX/47XXX carry mixed germ lines, which manifest as phenotypic abnormalities and POF (27). The association of POF and X chromosome deletions or X-autosomal translocations has been extensively reported in literature (15). Deletions of the X chromosome have been suggested in three main critical regions: X q13. 3- q22, Xq26-q28, as well as Xp11. 2 (28, 29). Deletions at Xp11. 2 result in 50% primary amenorrhea and 50% secondary amenorrhea, and deletions at X q13 usually produce primary amenorrhea (15). Balanced X chromosome and autosomal translocation have been reported in more than 100 POF cases, and the break points of balanced X-autosomal translocations were reported in the region between Xq13 and Xq27 (30).

Adolescent girls with Turner’s syndrome or other chromosomal abnormalities who still have follicles in their ovaries could be candidates for oocyte or/ and ovarian tissue cryopreservation, to save their ovaries for future fertilization by ovarian reimplantation (31, 32).

#### Fragile X syndrome

Fragile X syndrome is an X-linked dominant condition with incomplete penetration and a prevalence of 1/4000 in males and 1/6000 in females (33). It is the most common hereditary cause of mental retardation and developmental delay (34). It is caused by an expansion of CGG trinucleotide repeats in the 5′ untranslated region of the first exon of the fragile X mental retardation 1 (*FMR1*) gene. This gene is located on the X chromosome at Xq27.3. Fully affected individuals have more than 200 CGG repeats, whereas normally there are between 5 and 54 CGG repeats. Premutation alleles, defined as between 55 and 200 CGG repeats, are at risk of expanding into a full mutation in the next generation (35, 36). A recent study showed that a number of CGG repeats between 30 and 40 might be used to predict premature ovarian aging and POF in infertile patients (37). Expansion to more than 200 repeats leads to methylation-coupled silencing of the *FMR1* gene and absence of FMR-protein. This protein has an important role in prenatal and postnatal brain development (38).

The prevalence of the *FMR1* premutation carrier state in the general population of women is 1 in 100 (35), and approximately 16-26% of the female premutation carriers will develop POF (39,40). However, full-mutation carriers and their non-carrier sisters appear to have the same risk as seen in the general population (~1%) (41). The women who carry the *FMR1* premutation develop premature ovarian failure (42), tremor-ataxia syndrome (43), and mild neuro-cognitive dysfunction (36). Hundscheid et al. found that paternally inherited Fragile X premutations were more likely to give rise to POF than maternally inherited premutations. The authors hypothesized that an imprinting effect in POF could be confined to paternally inherited Fragile X premutation (44). This finding was not substantiated in subsequent studies (45, 46). Carriers of the *FMR1* premutation actually produce higher levels of *FMR1* m-RNA than normal, which causes neuronal cell degeneration (47). Some practitioners surmised that the same mechanism might be at work in the ovary as the cause of follicular atresia and ovarian failure in the carrier state (48, 49).

Approximately 14% of women with familial POF have a premutation in the *FMR1* gene, as compared to 2% of women with isolated POF (42). For women with sporadic spontaneous POF, no analysis of the cost/benefit or ethical, legal, or social implications of genetic counseling for Fragile X syndrome is available; however, the American College of Obstetrics and Gynecology (ACOG) recommended screening in these situations (33):

1. Individuals seeking reproductive counseling who have a family history of Fragile X syndrome or undiagnosed intellectual disability.
2. Fetuses of mothers or fathers with premutations or full mutations.
3. Young women with elevated levels of FSH hormone, especially with a family history of premature ovarian failure, Fragile X syndrome, or a relative of either sex with undiagnosed intellectual disability.

Actually, there is a need for each woman diagnosed with spontaneous POF to be informed of her increased risk of carrying a premutation in the *FMR1* gene and about the availability of genetic testing to detect this condition. Identification of families that carry the *FMR1* premutation would permit women of childbearing age to be counseled about their reproductive options and monitored more closely for the possible development of premature ovarian failure. PGD, CVS, and amniocentesis are options for prenatal diagnosis in situations with maternal premutation or full mutation (50).

The associations of POF and other abnormalities of the X chromosome have been extensively reported in literature (15). The breakpoint in Xq24 may be associated with POF (51). Bone morphogenetic protein 15 (*BMP15*) gene maps to Xp11. 2 within the Xp POF critical region. Heterozygous mutation of this gene was reported in two sisters with POF (52).

We found only one study in Iranian literature about chromosomal abnormalities in Iranian women with POF, which has showed definite abnormal X chromosomes in 17. 65% of these patients (27).

Abnormal karyotypes are detected in 13-50% of patients who develop primary or secondary amenorrhea due to POF. Thus, cytogenetic analysis should be performed as a part of basic evaluation of women diagnosed with POF. Having this information may influence the family planning decisions of family members (53). If Y chromosome material presents, gonadectomy is mandatory for the prevention of gonadoblastoma (53).

### Autosomal disorders

#### Galactosemia

Galactosemia is a rare autosomal recessive disorder which occurs due to a deficiency in the enzyme galactose-1-phosphate uridyltransferase (GALT). The GALT gene maps to chromosome 9p13. These patients develop hepatocellular, ocular, renal, and neurological damage as a result of the accumulation of galactose and its metabolites. The prevalence of POF is 60-70% in female patients with galactosemia (53). There is controversy about the pathophysiology of ovarian damage in galactosemia. It may be due to the toxic effect of galactose (or one of metabolites) on follicular structures, the decrease in the initial number of oogonia during fetal life, accelerated follicular atresia after birth and before puberty, defective gonadotropin function due to abnormalities in their carbohydrate composition and reduced bioactivity, and/or the neutral isoelectric point in FSH isoforms (53). So, the exact mechanism of ovarian failure has not been elucidated in patients with galactosemia and POF.

GALT 188Q is a genetic marker which has been identified in some patients with galactosemia. Premature ovarian dysfunction has not been diagnosed in individuals heterozygous for GALT188Q mutations. (54).

#### Blepharophimosis- ptosis- epichanthus inversus syndrome (BPES)

BPES is an autosomal dominant, sex-limited condition with a distinctive eyelid phenotype. Two forms have been described: in type I, POF related infertility is an adjunct to the condition, and type II is not associated with POF (55). BPES type I is mapped to 3q22-23(54). Two genes are identified within the breakpoint region. One of the genes, termed *FOXL2* appears predominantly in the ovaries of adult humans. In previous reports, all mutations had been exclusively localized in the *FOXL2* gene (56). However, two other members of this family, *FOXO1A* and *FOXO3A*, are candidate genes for the development of POF (57).

#### Autoimmune polyendocrinopathy- candidiasisectodermal dystrophy (APECED)

AIRE gene, is responsible for autoimmune polyendocrinopathy- candidiasis-ectodermal dystrophy syndrome (APECED) (53). This mutation, which is mapped to chromosome 21q23, can lead to hypogonadism and ovarian insufficiency (53).

#### Steroidogenic enzyme defect

Several congenital enzyme defects can disrupt estrogen synthesis; these defects result in low estrogen, delayed puberty, amenorrhea, and high serum FSH concentration levels despite the existence of normal-appearing primordial follicles in the ovary. Defects in the steroidogenic acute regulatory enzyme (StAR), CYP17, and aromatase enzymes cause these clinical and histological abnormalities (58, 59). Lack of appropriate negative feedback by peripheral estrogen on gonadotropins may lead to excessive follicular growth and increased risk of ovarian torsion and infarction in these hypoestrogenized patients (59).

#### Gonadotropin receptor dysfunction

FSH and luteinizing hormone (LH) have important roles in the recruitment, development, and maturation of ovarian follicles. FSH and LH receptor genes map to 2p21. Some studies have reported inactivating mutations of the FSH or LH receptor genes in connection with primary or secondary amenorrhea and hypergonadotropic ovarian failure (60, 61). Histological studies of ovaries in patients with FSH receptor gene mutations have showed a streak or hypoplastic gonad with impaired follicular development of the primordial and primary follicles. POF has been identified in patients with a defect in the guanine nucleotide regulatory protein of adenylate cyclase (G-protein), which is linked to the FSH and LH receptors as a second-messenger system. Due to the multiplicity of receptors activated by the same G-protein, pseudohypoparathroidism and hypothyroidism may be observed in these patients (62).

Breetherick et al. suggested that estrogen receptor-α (*ESR-1*) poly morphisms are associated with idiopathic POF; however they recommended further studies in larger patient samples to confirm this finding (63).

#### Inhibin gene mutation

The glycoprotein inhibin plays an important role in the recruitment and development of ovarian follicles by the negative feedback control of FSH. Inhibin α (*INHA*), Inhibin β-a (*INHBA*), and Inhibin β-b (*INHBB*) genes are responsible for inhibin coding. Mutation in the *INHA* gene was seen in POF patients (64-66). A recent study has shown that inhibin α-subunit (*INHA*) gene mutation is more frequent in patients with POF than in the normal fertile Iranian population (67).

#### Noggin mutation

Proximal symphalangism (SYM1) is an autosomal dominant disorder with characteristic skeletal abnormalities and conductive deafness. This disease has been shown to result from haploinsufficiency of the *NOG* gene on 17q22 encoding Noggin. *NOG* is expressed in the ovary and acts as an antagonist for BMPs, which play an important role in ovarian function (68). Kosaki et al (69). reported a case of POF and SYM1 with mutation in *NOG*. They proposed that *NOG* mutation may constitute one of the multiple susceptibility genes for the development of POF.

A recent study has shown that four polymorphisms in the protein L- isoaspartyl-0-methytransferase (*PCMTI*) gene, a protein repair enzyme, are associated with POF (70).

#### Autoimmune causes

Some cases of POF may be due to an abnormal self-recognition by the immune system. The exact mechanism remains obscure (71); probably the genetic or environmental factors initiate the immune response. The major histocompatibility complex antigen, cytokines, cell-mediated immunity and antibody-mediated immunity have important roles. Autoimmune mechanisms are involved in the pathogenesis of 4-30% of POF cases (10, 72). The evidences for an autoimmune etiology are:

1. Presence of lymphocytic oophoritis (72-74)
2. Demonstration of ovarian autoantibodies (74,75)
3. Associated autoimmune disorders (74) The oophoritis is characterized primarily by cellular infiltration of macrophages, natural killer cells, T-lymphocytes, plasma cells, and B- lymphocytes (73). The target of lymphocyte influx may be the class II MHC molecules on granulose cells (73).

Antiovarian antibodies in POF have been reported in several studies, but their specificity and pathogonomic roles are questionable. Some antigenic targets for antibody-mediated autoimmune damage in POF were identified:

1. Steroid producing cells (hillar cells, granulosa cells, theca internal and corpus luteum). Autoantibodies to steroid producing cells are widely present in POF associated with Addison’s disease (75). In autoimmune oophoritis, lymphatic infiltration is confined to secondary and antral follicles that have theca cells. This finding shows that steroid producing cells express the antigens that stimulate the immune response (74).
2. 3β-hydroxysteroid dehydrogenase (3β-HSD). This enzyme is involved in the steroid metabolic pathway. 3β-HSD autoantibodies were found in 2-21% patients with isolated POF (76, 77). However, Florni et al. showed that 3β-HSD is not a major autoantigen in autoimmune POF and suggested that this marker does not appear to be useful in routine clinical practice (75). The differences in the 3β-HSD antibody assay may in part explain the differences observed in these studies. The antibodies against steroidogenic enzymes such as 21- hydroxylase, 17- hydroxylase and side chain cleavage were seen in patients with histologically confirmed autoimmune oophoritis (72).
3. Gonadotropin receptor blocking antibodies. Tang et al. (78) have found a relation between POF and FSHR (FSH-receptor) antibodies in their patients, but in later published studies, the gonadotropin receptor blocking antibodies have not been detected in POF women (79). Thus gonadotropin receptor blocking antibodies are a rare causes of POF.
4. Other ovarian antigens. The oocyte, zona pellucida, and corpus luteum are other possible targets for autoantibody-induced damage (80, 81).

The published incidence of antiovarian antibodies in patients with POF ranges widely (7-67%) due to the heterogeneity of investigation methods, multiple ovarian antibody targets (including antibodies against steroidogenic enzymes, gonadotropins and their receptors, the corpus luteum, zona pellucida and oocytes), the transient appearance of antiovarian antibodies, different stages of disease, as well as variations in antibody test format and antigen presentation (15). Therefore interpretation of the role of antiovarian antibodies in POF is problematic. Antiovarian antibodies do not correlate with the presence or severity of oophritis and do not predict when ovarian failure will occur, so the measuring of these antibodies is not recommended. In addition, it is uncertain whether antiovarian antibodies are pathogenic or secondary to antigen release after cellular damage.

Approximately 3% of women with POF have an associated endocrine dysfunction known as autoimmune polyglandular syndrome (APS), types I and II. The type I syndrome is a rare autosomal recessive disorder in young children, characterized by multiple organ-specific autoimmunity secondary to a variety of autoantibodies directed against key intracellular enzymes. POF in the form of primary amenorrhea develops in 60% of these patients. APS type II is an autosomal dominant disorder, and is associated with gonadal failure in 4% of patients (82). Addison’s disease is a component of both ASP types. Approximately 10% of women with Addison’s have POF (73) and 2-10% of POF cases show evidence of autoimmunity against the adrenal (71, 83). Sharing of autoantigens between ovary and adrenal glands, particularly the P450 side-chain cleavage enzyme, may explain the association of ovarian failure with Addison’s disease. In subclinical patients (presence of adrenal antibodies in absence of hypocortisolism), Addison’s disease may develop by the age of 8-14 (83).

Circulating 21-hydroxylase antibodies are the best immune markers of autoimmune adrenal insufficiency (75). Testing for anti-adrenal and anti-21-hydroxylase antibodies by indirect immunofluorescence not only identifies POF-diagnosed women who have steroidogenic cell autoimmunity and occult adrenal insufficiency at the time of initial presentation, but also identifies patients who should be monitored for the subsequent development of adrenal insufficiency, a potentially fatal disorder (15, 75, 83). Bakalov et al. showed that morning serum cortisol levels have low sensitivity and specificity, and ACTH stimulation tests have a high false positive rate when used as screening tests in these patients (83). Thus, the ACTH stimulation test should be used as a diagnostic test for patients with symptoms of adrenal insufficiency or with positive adrenal autoantibodies. Women with positive adrenal antibodies will require referral to a medical endocrinologist for additional evaluation and long-term follow-up of their adrenal function.

Several other autoimmune disorders have been associated with POF; hypothyroidism is the most common. Both endocrine (thyroid, hypoparathyroid, diabetes mellitus, hypophsitis) and non-endocrine disorders (chronic candidiasis, idiophatic thrombocytopenic purpura, vitiligo, alopecia, autoimmune hemolytic anemia, pernicious anemia, SLE, rheumatoid arthritis, Crohn’s disease, Sjogren syndrome, primary billiary cirrhosis and chronic active hepatitis) were observed in association with autoimmune POF (84).

Ashrafi et al. (85) found the presence of anti-thyroid and anti-ovarian antibodies in a familiar type of POF, so they proposed a genetic component for developing autoimmune POF. In our opinion, presence of antiovarian antibodies in a familial type of POF could be secondary to cellular damage or changes being followed by ovarian dysfunction; therefore, we recommend further studies in larger patient samples to confirm their finding.

Autoimmune hypothyroidism is a disease commonly associated with POF, so screening by the measurement of TSH, free T4, anti-thyroid-peroxidase and anti-thyroglobuline antibodies levels is recommended.

The gold standard for detecting autoimmune POF is ovarian biopsy, which is not recommended due to unknown clinical value, expense, and risks (15). The predictive value of commercially available antiovarian antibody tests is poor, so there is no validated serum marker to identify patients with autoimmune POF (15, 86). However, Yan et al. suggested that a significant increase in CD8 density on T cells could be a reliable indicator of the involvement of the immune system in POF (87).

In autoimmune ovarian failure, the elevated serum gonadotropin hormones result from the dysfunction of the ovarian follicles rather than follicular depletion. Also, the lymphatic infiltration is confined to secondary and antral follicles while primordial follicles are spared, so resumption of ovarian function with or without conception can be found in a substantial number of affected patients (88). Theoretically, ovarian function might be restored if a safe and effective immunosuppressive regimen could be used, but the placebo-controlled randomized clinical trials, using corticosteroids as immunosuppressive therapy, failed to show a change in the course of the disease (73). In addition, the use of corticosteroids as immunomodulatory treatment may cause more harm than good; ostenecrosis secondary to glucocorticoid therapy in POF has been reported (89).

#### Iatrogenic causes

Chemotherapy and radiotherapy can lead to POF in patients developing malignant disease (90-92). Iatrogenic POF has increased over time, because of the improved success in the treatment of cancer in children, adolescents, and reproductive-age women and the increase in the practice of prophylactic bilateral oopherectomy at the time of hysterectomy (93). The radiosensitivity of the oocyte is estimated to be 2 Gray (94), and an ovarian radiation dose of ≥6 Gray produces ovarian failure in virtually all individuals over 40 years of age (95). The effect of radiotherapy is dependent on dose and age, and on the radiation therapy field. Lateral transposition of the ovaries, out of the field of irradiation, in young women requiring pelvic irradiation helps in preserving their ovarian function.

The exact mechanisms involved in chemotherapyinduced ovarian failure are not known; however the functions of granulosa cells and oocytes may be affected by chemotherapeutic agents (96). The age of the individual, the drug type (use of alkylating agents) and dosage, and the addition of radiation to the treatment are the major predictive factors in the development of ovarian dysfunction after chemotherapy (91). The prepubertal patients are relatively resistant to this effect of chemotherapy (97). The serum anti-mullerian hormone, as a biochemical marker of ovarian reserve, may be useful for evaluating the gonadotoxicity of chemotherapy regimens (98).

Although several investigators have demonstrated that GnRH agonist may inhibit chemotherapyinduced ovarian failure in animal models, it is a controversial issue in humans (99-101). The possible explanatory mechanisms of GnRH agonist in decreasing the chemotherapy-associated gonadotoxicity could be: decrease in FSH level, direct effect on ovary independent of the suppressive effect on gonadotropin levels, increase in intragonadal antiapoptotic molecules, decrease in utero-ovarian perfusion owing to a hypoestrogenic state, and decreased exposure of the ovaries to the chemotherapeutic agents (101). In an animal model study, GnRH antagonist did not protect the ovary from the damaging effect of cyclophosphamide (102). Oral contraceptives (OC) during chemotherapy do not protect ovarian function in patients receiving high-dose chemotherapy (103).

Ovarian suppression by GnRH agonist was offered to every referred female patient before chemotherapy for malignant diseases (99, 100, 101). The Practice Committee of the American Society for Reproductive Medicine (ASRM) concluded that only the efficiency of IVF-ET, and embryo cryopreservation, were proven in women undergoing chemotherapy (104). The other interventions including GnRH analog suppression, use of estradiol or glucocorticoids, ovarian hyperstimulation, and oocyte or ovarian tissue cryopreservation, are still considered investigational (105) by ASRM (104).

Ovarian or egg cryopreservation and transplantation of the thawed tissue are not yet clinically established; however there is no contraindication for cryopreservation combined with GnRH agonist administration (101).

Some studies have reported the spontaneous return of ovarian function after several years in women with chemotherapy- or radiotherapy-induced ovarian failure (106, 107). The exact mechanism is unknown; however, it is theoretically possible that the constant stimulation of ovaries by postmenopausal levels of gonadotropins may have resulted in follicle development. Predictive factors for ovarian recovery include younger age at first chemotherapy administration and absence of concomitant radiotherapy (91). Successful pregnancies have been reported among women who had return of ovarian function. There is an increase in miscarriage, small for gestational age offspring, and reduction in live births in women treated with chemotherapy (108).

Although no prospective studies of ovarian function and gonadotropin levels before and after pelvic-adnexal surgeries have been done, these procedures have the potential to damage the ovary by affecting its blood supply or causing inflammation in the pelvic area (109,110). Recovery after interventions that compromise ovarian blood supply would seem to be possible if sufficient collateral circulation develops and the resting follicles resume their cycles.

Uterine artery embolization has a potential to result in POF by compromising the vascular supply to the ovary (109, 111). It is unknown whether polyvinyl alcohol particles used for embolization have a direct toxic effect on the ovary.

#### Infectious causes

Mumps oophoritis has been considered to be a cause of POF. True incidence of post-oophritis ovarian failure is unknown. In the vast majority of affected women, return of ovarian function occurs following recovery (1, 15).

There are also anecdotal reports of viral and microbial infection, such as tuberculosis, varicella, cytomegalovirus, malaria, and shigella being followed by POF (12).

#### Environmental toxins

Smoking is the most widely studied toxin that alters ovarian function, and on average, the female smokers experience menopause earlier than nonsmokers suggesting a possible detrimental effect of cigarette smoking on ovarian function (112). Chang et al. (113) reported an increased risk of idiopathic POF with cigarette smoking. An increased risk of developing POF has also been reported in the women with epilepsy (114).

## Conclusion

POF is defined by the association of amenorrhea, sex steroid deficiency and menopausal levels of serum gonadotropins before the age of 40 years. Intermittent and unpredictable ovarian function, and even spontaneous pregnancy have been reported in young women with spontaneous POF subsequent to diagnosis (4, 7). “Premature ovarian dysfunction'' or ''premature ovarian insufficiency'' may be a more accurate phrase to reflect the reversible nature of this condition.

Although most cases of POF are idiopathic (13), diverse etiologies are associated with POF (Table 1). The observation of familial cases with POF indicates the role of genetic aberrations in its pathogenesis (14). Genetic defects mostly involve the X chromosome. X chromosome abnormalities range from a numerical defects like complete deletion of one X chromosome to partial defects in the form of deletions, isochromosomes and balanced X autosome translocations (16). Association between 48XXXX, Trisomy X, X chromosome deletions, X-autosomal translocations, mosaic conditions such as 45X/46XX, 46XX/47XXX, and POF have been extensively reported in literature (15, 16, 24, 26, 27).

Adolescent girls with Turner’s syndrome or other chromosomal abnormalities who still have follicles in their ovaries could be candidates for oocyte or/ and ovarian tissue cryopreservation, to save their ovaries for future fertilization (31, 32).

Fragile X syndrome is an X-linked dominant condition with incomplete penetration, (33). It is the most common hereditary cause of mental retardation and developmental delay (34). Premutation alleles are at risk of expansion into a full mutation in the next generation (35, 36). A recent study showed that a number of CGG repeats between 30 and 40 might be used to predict premature ovarian aging and POF in infertile patients (37). The women who carry the *FMR1* premutation develop premature ovarian failure (42), tremor-ataxia syndrome (43), and mild neuro-cognitive dysfunction (36).

Carriers of the *FMR1* premutation actually produce higher levels of *FMR1* m-RNA than normal, which might cause ovarian damage (48, 49).

There is a need for each woman diagnosed with spontaneous POF to be informed of her increased risk for carrying a premutation in the *FMR1* gene and about the availability of genetic testing to detect this condition. Identification of families that carry the *FMR1* premutation would permit women of childbearing age to be counseled about their reproductive options and monitored more closely for the possible development of premature ovarian failure. PGD, CVS, and amniocentesis are options for prenatal diagnosis in situations with maternal premutation or full mutation (50).

An increasing number of studies have documented autosomal involvement in ovarian dysfunction. Heterozygous mutation of the bone morphogenetic protein 15 (*BMP15*) gene has been reported in two sisters with POF (52). Galactosemia is a rare autosomal recessive disorder which arises due to a deficiency in the enzyme GALT (53). The pathophysiology of ovarian damage in this condition could be due to the toxic effect of galactose (or one of the metabolites) on follicular structures or defective gonadotropin function and structure. The exact mechanism of ovarian failure has not been elucidated in patients with galactosemia and POF.

POF related infertility is an adjunct to BPES type I. In previous reports, all mutations were exclusively localized in the *FOXL2* gene (56). However, in recent studies, it was discovered that two other members of this family, *FOXO1A* and *FOXO3A*, are candidate genes for the development of POF (57). Mutation in AIRE gene has been identified in patients with hypogonadism and ovarian insufficiency (53).

Defects in the steroidogenic acute regulatory enzyme (StAR), CYP17, and aromatase enzymes cause primary amenorrhea and POF (58, 59). Lack of appropriate negative feedback by peripheral estrogen on gonadotropins may lead to excessive follicular growth and increased risk of ovarian torsion and infarction in these hypoestrogenized patients (59).

Some studies reported inactivating mutations of the FSH or LH receptor genes in connection with primary or secondary amenorrhea and hypergonadotropic ovarian failure (60, 61). Polymorphisms in estrogen receptor -α (*ESR-1*) and L- isoaspartyl- 0-methytransferase (*PCMTI*) genes are associated with idiopathic POF (63, 70).

A recent study among the Iranian population has showed that inhibin α-subunit (*INHA*) gene mutation is more frequent in patients with POF than in the normal fertile Iranian population (67).

*NOG* mutation may constitute one of the multiple susceptibility genes for the development of POF (69).

Karyotypes should be performed as a part of basic evaluation of women diagnosed with POF due to frequent abnormal karyotypes (13-50%) in this condition. Having this information may influence the family planning decisions of family members (53). If Y chromosome material presents, gonadectomy would be mandatory for the prevention of gonadoblastoma (53).

Some cases of POF may be due to an abnormal self-recognition by the immune system. The exact mechanism remains obscure (71). Presence of lymphocytic oophoritis (72, 73, 74), demonstration of ovarian autoantibodies (73, 74), and association with other autoimmune disorders (75) are evidences of the presence of autoimmune mechanisms in the pathophysiology of POF.

Antiovarian antibodies in POF have been reported in several studies, but their specificity and pathogonomic roles are questionable. Antiovarian antibodies do not correlate with the presence or severity of oophritis, so the measurement of these antibodies is not recommended. The measuring of CD8 density on T cells could provide a reliable indicator of the involvement of the immune system in POF (87).

Autoimmune Polyglandular Syndrome (APS) type I is a rare autosomal recessive disorder in young children, characterized by multiple organ-specific autoimmunity secondary to a variety of autoantibodies directed against key intracellular enzymes. POF in the form of primary amenorrhea develops in 60% of these patients.

Autoimmunity against the adrenal gland has been shown in 2-10% of POF cases (71, 83). Sharing of autoantigens between ovary and adrenal glands, particularly the P450 side-chain cleavage enzyme, may explain the association of ovarian failure and Addison’s disease. In subclinical patients (presence of adrenal antibodies in absence of hypocortisolism), Addison’s disease may develop by the age of 8-14 (83). Circulating 21-hydroxilase antibodies are the best immune marker of autoimmune adrenal insufficiency (75). Testing for anti-adrenal and anti-21-hydroxylase antibodies are the best biochemical tests for the detection of steroidogenic cell autoimmunity and occult adrenal insufficiency, and identification of the patients who should be monitored for the subsequent development of adrenal insufficiency (15, 75, 83). Morning serum cortisol level has low sensitivity and specificity, when used as a screening test in these patients (83). The ACTH stimulation test should be used as a diagnostic test for patients with symptoms of adrenal insufficiency or with positive adrenal autoantibodies. Women with positive adrenal antibodies will require referral to a medical endocrinologist for additional evaluation and long-term follow-up of their adrenal function.

Autoimmune hypothyroidism is the disease most commonly associated with POF, so screening by measurement of TSH, free T4, anti-thyroid-peroxidase and anti-thyroglobuline antibody levels is recommended.

The gold standard for detecting autoimmune POF is ovarian biopsy, which is not recommended due to unknown clinical value, expense, and risks (15).

Placebo-controlled randomized clinical trials, using corticosteroids as immunosuppressive therapy, failed to show a change in the course of autoimmune POF (73).

Iatrogenic POF has increased over time (93). The major predictive factors in the development of ovarian dysfunction after chemotherapy and/or radiotherapy are age, dosage and drug type, radiation therapy field, and concomitant therapy. (91).

The serum anti-mullerian hormone, as a biochemical marker of ovarian reserve, may be useful for evaluating the gonadotoxicity of chemotherapy regimens (98).

The usefulness of GnRH agonist for inhibiting chemotherapy-induced ovarian failure is a controversial issue in humans (99-101). In several studies, GnRH antagonist and OC did not protect the ovary from the damaging effect of chemotherapy (102,103).

Some studies have reported the spontaneous return of ovarian function after several years in women with chemotherapy- or radiotherapy-induced ovarian failure (106, 107).

Predictive factors for ovarian recovery after chemotherapy- or radiotherapy-induced ovarian failure include younger age at first chemotherapy administration and absence of concomitant radiotherapy (91).

There is an increase in miscarriage, small for gestational age offspring, and reduction in live births in women treated with chemotherapy (108).

Uterine artery embolization has a potential for POF by compromising the vascular supply of the ovary (109,111).

Mumps oophoritis has been considered to be a cause of POF. Return of ovarian function has been shown in the vast majority of affected patients (1, 15). There are anecdotal reports of POF after other viral and microbial infections (12). The increased risk of idiopathic POF with cigarette smoking has also been reported (113).

In conclusion, although in the majority of POF cases the underlying cause is not identified, several etiological factors can affect normal ovarian function and lead to permanent or transient ovarian failure. Thorough screening for associated autoimmune disorders and cytogenetic analysis should be performed as a part of the diagnostic work-up. There is no value in ovarian biopsy for diagnosis.
